# Identification of key pathways and genes in response to trastuzumab treatment in breast cancer using bioinformatics analysis

**DOI:** 10.18632/oncotarget.24605

**Published:** 2018-03-05

**Authors:** Fanxin Zeng, Jiangping Fu, Fang Hu, Yani Tang, Xiangdong Fang, Fanwei Zeng, Yanpeng Chu

**Affiliations:** ^1^ Institute of Molecular Medicine, Peking University, Beijing, China; ^2^ Dazhou Central Hospital Clinic Medical Center, Dazhou, Sichuan, China; ^3^ Department of Oncology, Dazhou Central Hospital, Dazhou, Sichuan, China

**Keywords:** bioinformatics analysis, breast cancer, microarray, differentially expressed gene, trastuzumab

## Abstract

Breast cancer (BC) is one of the leading causes of death among women worldwide. The gene expression profile GSE22358 was downloaded from the Gene Expression Omnibus (GEO) database, which included 154 operable early-stage breast cancer samples treated with neoadjuvant capecitabine plus docetaxel, with (34) or without trastuzumab (120), to identify gene signatures during trastuzumab treatment and uncover their potential mechanisms. The gene ontology (GO) and Kyoto Encyclopedia of Genes and Genomes pathway (KEGG) enrichment analyses were performed, and a protein–protein interaction (PPI) network of the differentially expressed genes (DEGs) was constructed by Cytoscape software. There were 2284 DEGs, including 1231 up-regulated genes enriched in DNA replication, protein N-linked glycosylation via asparagine, and response to toxic substances, while 1053 down-regulated genes were enriched in axon guidance, protein localization to plasma membrane, protein stabilization, and protein glycosylation. Eight hub genes were identified from the PPI network, including GSK3B, RAC1, PXN, ERBB2, HSP90AA1, FGF2, PIK3R1 and RAC2. Our experimental results showed that GSK3B was also highly expressed in breast cancer tissues and was associated with poor survival, as was β-catenin. In conclusion, the present study indicated that the identified DEGs and hub genes further our understanding of the molecular mechanisms underlying trastuzumab treatment in BC and highlighted GSK3B, which might be used as a molecular target for the treatment of BC.

## INTRODUCTION

Breast cancer (BC) is one of the leading causes of death among women worldwide [1]. The increasing incidence of BC is due to various genetic and environmental changes that lead to the disruption of the cellular signaling network [2–5]. In 2012, there were 14.1 million new cancer cases globally, and BC accounted for 11.8% of them [6]. The introduction of trastuzumab, a humanized monoclonal antibody targeting the extracellular domain of HER2, revolutionized the treatment of HER2-positive BC [7–9]. The current gold standard in clinical practice is 1 year of adjuvant trastuzumab administration. Several trials have been conducted, looking to further refine the adjuvant treatment of patients with early-stage HER2-positive BC.

Catenin beta-1, also known as β-catenin, is a protein that is encoded by the *CTNNB1* gene. β-Catenin is an oncogene that plays a key role in the signaling output of the canonical Wnt cascade [10]. Wnt signaling results in β-catenin accumulation and transcriptional activation of specific target genes that regulate a remarkable variety of cellular processes, such as cell proliferation, cell survival and migration [11]. Mutations and overexpression of β-catenin are associated with many cancers, including hepatocellular carcinoma, colorectal carcinoma, lung cancer, breast tumors, ovarian cancer and endometrial cancer [12]. Although mutation of CTNNB1 is rare in BC [13], mounting evidence has revealed that the mutations in CTNNB1 are often associated with an upregulation of β-catenin and the pathogenesis of endometrial cancer and ovarian cancer [14]. Several variants of CTNNB1 were found to be associated with BC risk [15, 16], but the mechanism of CTNNB1 in BC is still unknown.

Glycogen synthase kinase 3 beta, also known as GSK3B, is an enzyme that in humans is encoded by the *GSK3B* gene. GSK3B, a substrate of PI3K/Akt signaling, plays an important role in fundamental functions, such as the cell cycle, cytoskeletal integrity, apoptosis, transcription factor expression and formation of neurofibrillary tangles through PI3K/Akt signaling [17, 18]. GSK3B has also been shown to interact with CTNNB1 [19], as the phosphorylation of GSK3B leads to β-catenin nuclear accumulation [20].

There are no reliable biomarker profiles available to identify key genes and pathways in BC with trastuzumab treatment. Furthermore, numerous clinical studies have been performed with data (GSE22358) from the Gene Expression Omnibus (GEO, www.ncbi.nlm.nih.gov/geo/) [21–25]. The Glück *et al.* [26] dataset (GSE22358) includes 154 stage II–III samples from patients with operable early-stage breast cancer prior to neoadjuvant chemotherapy of capecitabine plus docetaxel, with (34) or without (120) trastuzumab. The data include histologic grade, molecular subtype, ER-status, PR-status, HER2-status, p53 status and response to treatment. Therefore, we chose GSE22358 to identify a molecular predictor of trastuzumab benefit in BC.

In this study, we chose GSE22358 from GEO and used the GEO2R online tool to detect the differentially expressed genes (DEGs). Subsequently, the DEGs were screened using Gene-E software and hub genes with a high degree of connectivity were selected. Next, we established a PPI network of the DEGs gene ontology (GO) and pathway enrichment analysis. Moreover, analyses of biological process (BP), molecular function (MF), cellular component (CC) and KEGG pathways of the DEGs and three modules were performed. Overall survival (OS) analysis of these hub genes was performed using the Kaplan-Meier plotter online database (http://kmplot.com/analysis/). By analyzing the biological functions and pathways, we may gain further insight into BC treatment at a molecular level and explore the potential candidate biomarkers for diagnosis, prognosis, and drug targets.

## RESULTS

### Identification of DEGs

**Figure 1:**
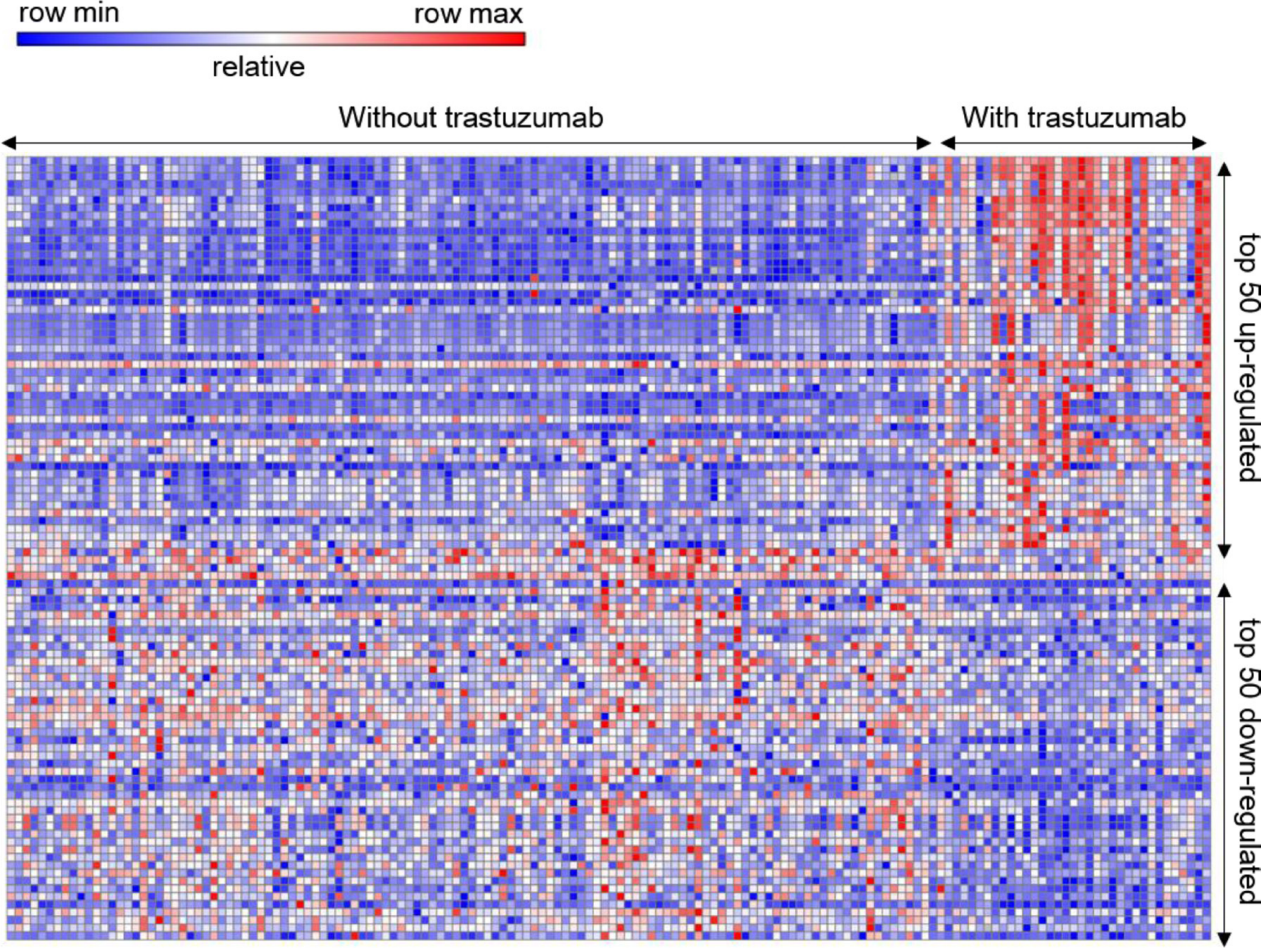
Heat map of the top 100 differentially expressed genes Hierarchical clustering analysis to categorize the data into two groups that had similar expression patterns with or without trastuzumab Red: up-regulation, *N* = 50; Purple: down-regulation, *N* = 50.

A total of 154 operable early-stage BC samples receiving neoadjuvant capecitabine plus docetaxel, with (34) or without trastuzumab (120) were analyzed. The series from each chip was analyzed separately using GENE-E software, which identified the DEG lists. Based on the GENE-E analysis, using *P* < 0.05 criteria, a total of 2284 genes were identified after the analyses of GSE22358 of which 1231 were up-regulated and 1053 were down-regulated. The DEG expression heat map (top 50 up-regulated and down-regulated genes) is shown in Figure 1. We uploaded all DEGs to the online software DAVID to identify overrepresented GO categories and KEGG pathways. GO analysis results showed that up-regulated DEGs were significantly enriched in biological processes (BP), including DNA replication, protein N-linked glycosylation via asparagine, response to toxic substance, mRNA 3′-end processing, and anaphase-promoting complex-dependent catabolic process (Table 1); the down-regulated DEGs were significantly enriched in biological processes, including axon guidance, protein localization to plasma membrane, protein stabilization, protein glycosylation, and regulation of phosphatidylinositol 3-kinase signaling (Table 1). For molecular function (MF), the upregulated DEGs were enriched in DNA polymerase binding, ATPase activity, protein binding, microtubule binding, and poly(A) RNA binding, and the down-regulated DEGs were enriched in solute:proton symporter activity, metallocarboxypeptidase activity, ErbB-3 class receptor binding, calcium-dependent protein binding, and protein tyrosine phosphatase activity (Table 1). In addition, GO cell component (CC) analysis showed that the up-regulated DEGs were significantly enriched in the nucleoplasm, membrane, melanosome, nucleus, and intracellular membrane-bound organelles, and down-regulated DEGs enriched in the Golgi membrane, endoplasmic reticulum membrane, membrane, Golgi apparatus, and actin cytoskeleton (Table 1).

**Table 1:** Gene ontology analysis of differentially expressed genes associated with trastuzumab

Expression	Category	Term	Count	%	*P*-Value
Up-regulated	GOTERM_BP_DIRECT	DNA replication	25	2	8.80E-05
	GOTERM_BP_DIRECT	protein N-linked glycosylation via asparagine	11	0.9	1.80E-04
	GOTERM_BP_DIRECT	response to toxic substance	16	1.3	1.10E-03
	GOTERM_BP_DIRECT	mRNA 3’-end processing	11	0.9	1.20E-03
	GOTERM_BP_DIRECT	anaphase-promoting complex-dependent catabolic process	14	1.1	1.70E-03
	GOTERM_BP_DIRECT	DNA damage response, signal transduction by p53 class mediator resulting in cell cycle arrest	12	1	2.00E-03
	GOTERM_BP_DIRECT	cell division	38	3.1	2.00E-03
	GOTERM_BP_DIRECT	mRNA splicing, via spliceosome	27	2.2	2.70E-03
	GOTERM_BP_DIRECT	regulation of signal transduction by p53 class mediator	18	1.5	2.80E-03
	GOTERM_BP_DIRECT	DNA repair	28	2.3	2.90E-03
	GOTERM_MF_DIRECT	DNA polymerase binding	6	0.5	4.00E-04
	GOTERM_MF_DIRECT	ATPase activity	25	2	9.80E-04
	GOTERM_MF_DIRECT	protein binding	449	36.5	2.50E-03
	GOTERM_MF_DIRECT	microtubule binding	25	2	4.70E-03
	GOTERM_MF_DIRECT	poly(A) RNA binding	96	7.8	6.50E-03
	GOTERM_MF_DIRECT	damaged DNA binding	11	0.9	6.70E-03
	GOTERM_MF_DIRECT	histone deacetylase binding	15	1.2	8.60E-03
	GOTERM_MF_DIRECT	helicase activity	13	1.1	1.00E-02
	GOTERM_MF_DIRECT	sulfuric ester hydrolase activity	5	0.4	1.40E-02
	GOTERM_MF_DIRECT	ATP binding	120	9.8	1.50E-02
	GOTERM_CC_DIRECT	nucleoplasm	238	19.3	2.70E-07
	GOTERM_CC_DIRECT	membrane	181	14.7	1.00E-04
	GOTERM_CC_DIRECT	melanosome	18	1.5	1.80E-04
	GOTERM_CC_DIRECT	nucleus	395	32.1	3.00E-04
	GOTERM_CC_DIRECT	intracellular membrane-bounded organelle	56	4.6	5.50E-04
	GOTERM_CC_DIRECT	cytosol	256	20.8	8.50E-04
	GOTERM_CC_DIRECT	kinetochore	14	1.1	1.20E-03
	GOTERM_CC_DIRECT	nucleolus	76	6.2	2.70E-03
	GOTERM_CC_DIRECT	proteasome complex	11	0.9	4.50E-03
	GOTERM_CC_DIRECT	condensed chromosome kinetochore	13	1.1	7.40E-03
	GOTERM_BP_DIRECT	axon guidance	20	1.9	1.40E-03
	GOTERM_BP_DIRECT	protein localization to plasma membrane	11	1	1.70E-03
	GOTERM_BP_DIRECT	protein stabilization	17	1.6	3.00E-03
	GOTERM_BP_DIRECT	protein glycosylation	15	1.4	3.00E-03
	GOTERM_BP_DIRECT	regulation of phosphatidylinositol 3-kinase signaling	12	1.1	3.40E-03
	GOTERM_BP_DIRECT	response to cocaine	8	0.8	3.60E-03
	GOTERM_BP_DIRECT	ERBB2 signaling pathway	8	0.8	4.20E-03
	GOTERM_BP_DIRECT	substrate adhesion-dependent cell spreading	8	0.8	4.20E-03
	GOTERM_BP_DIRECT	centrosome localization	5	0.5	4.30E-03
	GOTERM_BP_DIRECT	O-glycan processing	10	0.9	5.20E-03
	GOTERM_MF_DIRECT	solute:proton symporter activity	3	0.3	8.40E-03
	GOTERM_MF_DIRECT	metallocarboxypeptidase activity	6	0.6	1.40E-02
	GOTERM_MF_DIRECT	ErbB-3 class receptor binding	3	0.3	1.60E-02
	GOTERM_MF_DIRECT	calcium-dependent protein binding	9	0.9	1.60E-02
	GOTERM_MF_DIRECT	protein tyrosine phosphatase activity	12	1.1	1.90E-02
Down-regulation	GOTERM_MF_DIRECT	receptor signaling protein activity	7	0.7	2.00E-02
	GOTERM_MF_DIRECT	protein phosphatase binding	9	0.9	2.00E-02
	GOTERM_MF_DIRECT	protein kinase binding	31	2.9	2.20E-02
	GOTERM_MF_DIRECT	zinc ion binding	81	7.7	2.50E-02
	GOTERM_MF_DIRECT	1-phosphatidylinositol-3-kinase activity	7	0.7	2.70E-02
	GOTERM_CC_DIRECT	Golgi membrane	58	5.5	1.70E-05
	GOTERM_CC_DIRECT	endoplasmic reticulum membrane	77	7.3	2.50E-05
	GOTERM_CC_DIRECT	membrane	152	14.4	1.10E-03
	GOTERM_CC_DIRECT	Golgi apparatus	67	6.4	3.30E-03
	GOTERM_CC_DIRECT	actin cytoskeleton	22	2.1	8.50E-03
	GOTERM_CC_DIRECT	cytoplasm	317	30.1	1.10E-02
	GOTERM_CC_DIRECT	mitochondrial membrane	12	1.1	1.20E-02
	GOTERM_CC_DIRECT	axon	22	2.1	1.70E-02
	GOTERM_CC_DIRECT	cis-Golgi network	7	0.7	1.80E-02
	GOTERM_CC_DIRECT	uropod	4	0.4	1.90E-02

Abbreviations: GO: Gene Ontology.

### KEGG pathway analysis

Table 2 contains the most significantly enriched pathways of the up-regulated DEGs and down-regulated DEGs analyzed by KEGG analysis. The up-regulated DEGs were enriched in protein processing in the endoplasmic reticulum, base excision repair, pentose and glucuronate interconversions, proteasome, and ascorbate and aldarate metabolism while the down-regulated DEGs were enriched in proteoglycans in cancer, the ErbB signaling pathway, bacterial invasion of epithelial cells, the insulin signaling pathway, and the neurotrophin signaling pathway.

**Table 2:** KEGG pathway analysis of differentially expressed genes associated with trastuzumab

Category	KEEG Term	Count	%	*P*-Value
Down-regulation	Proteoglycans in cancer	23	2.2	1.40E-03
	ErbB signaling pathway	13	1.2	2.80E-03
	Bacterial invasion of epithelial cells	12	1.1	3.40E-03
	Insulin signaling pathway	17	1.6	3.70E-03
	Neurotrophin signaling pathway	15	1.4	6.00E-03
Up-regulation	Protein processing in endoplasmic reticulum	27	2.2	1.10E-04
	Base excision repair	8	0.7	6.90E-03
	Pentose and glucuronate interconversions	8	0.7	1.10E-02
	Proteasome	8	0.7	3.20E-02
	Ascorbate and aldarate metabolism	6	0.5	3.70E-02

Abbreviations: KEGG: Kyoto Encyclopedia of Genes and Genomes.

### Module screening from the PPI network

**Figure 2:**
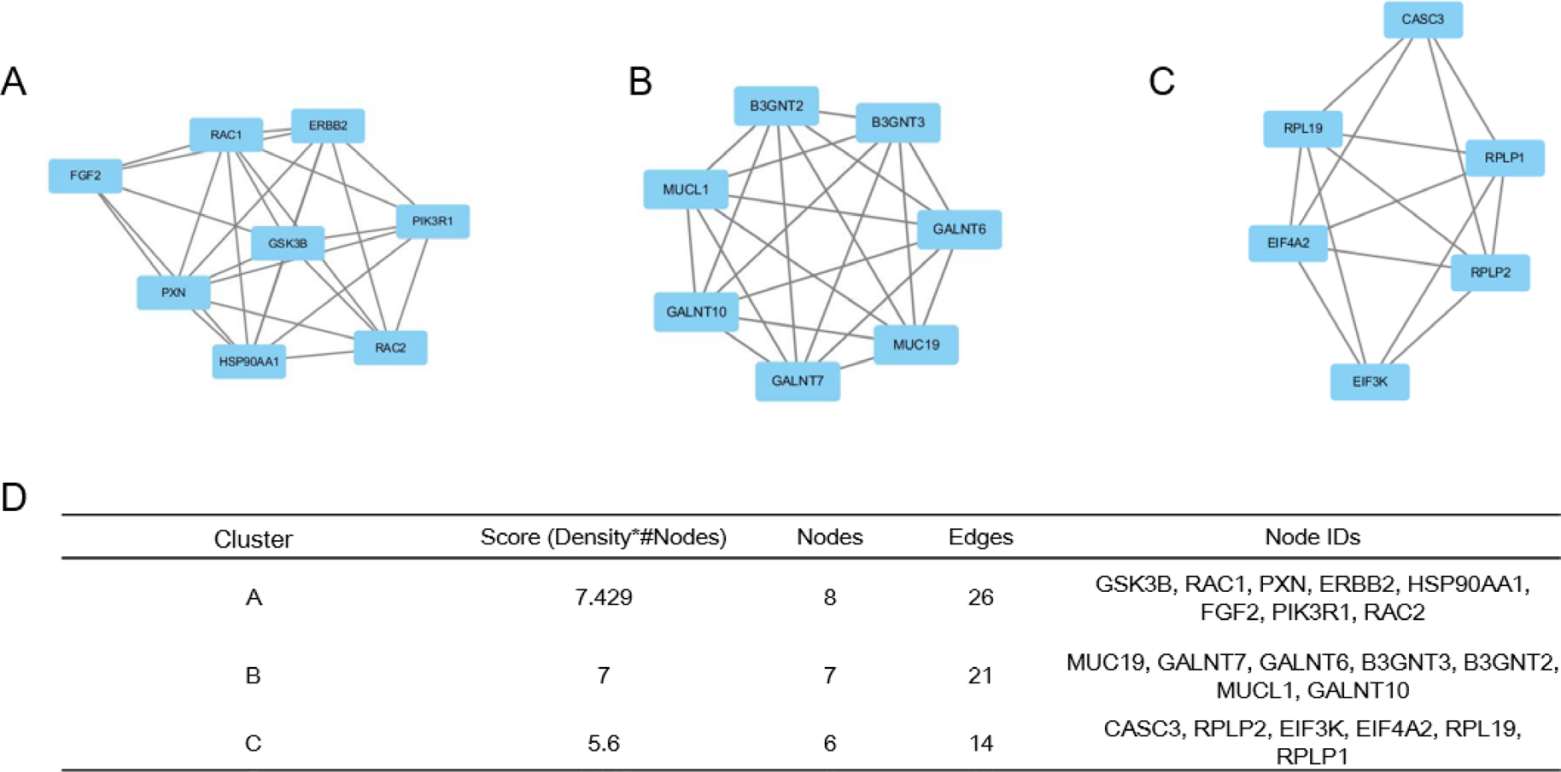
Top 3 modules from the protein–protein interaction network (**A**) module 1, (**B**) module 2, (**C**) module 3 were analyzed using the plug-in Molecular Complex Detection in Cytoscape based on the information in the STRING database, (**D**) the enriched pathways and functional annotation of the genes involved in the modules of 1, 2, and 3.

Based on the information in the STRING database, among these genes, GSK3B showed a 42-node degree. Moreover, a total of 1000 nodes and 2079 edges were analyzed using the plug-in MCODE. The top 3 significant modules were selected, and the functional annotation of the genes involved in the modules were analyzed (Figure 2). Enrichment analysis showed that the genes in module 1 were mainly associated with GSK3B, RAC1, PXN, ERBB2, HSP90AA1, FGF2, PIK3R1, and RAC2.

### GSK3B is upregulated in breast cancer

**Figure 3:**
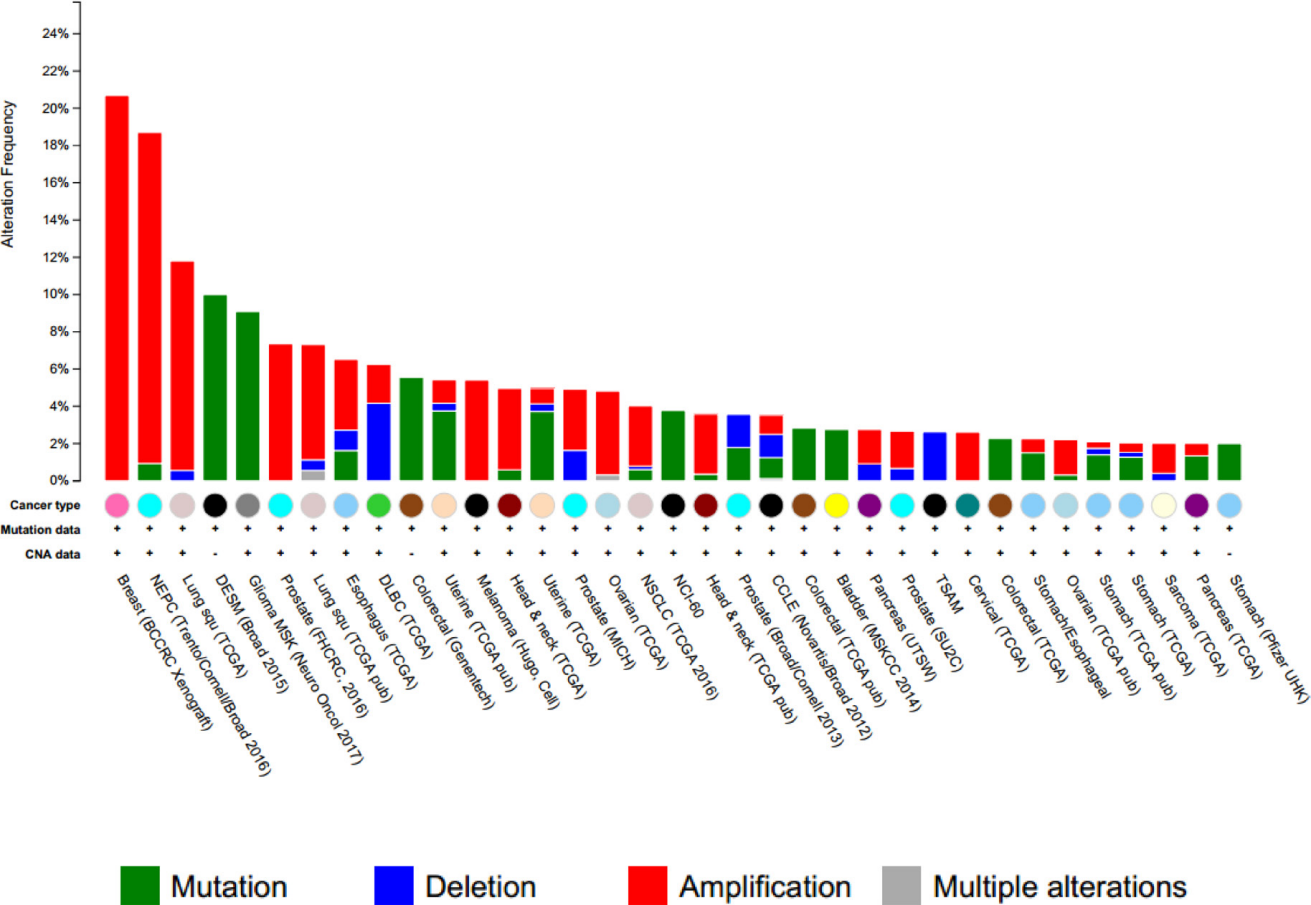
Cross-cancer alteration summary for GSK3B (166 studies/1 gene) GSK3B was analyzed from the cBioPortal for Cancer Genomics (http://www.cbioportal.org). The red column indicates the amplification pattern.

**Figure 4:**
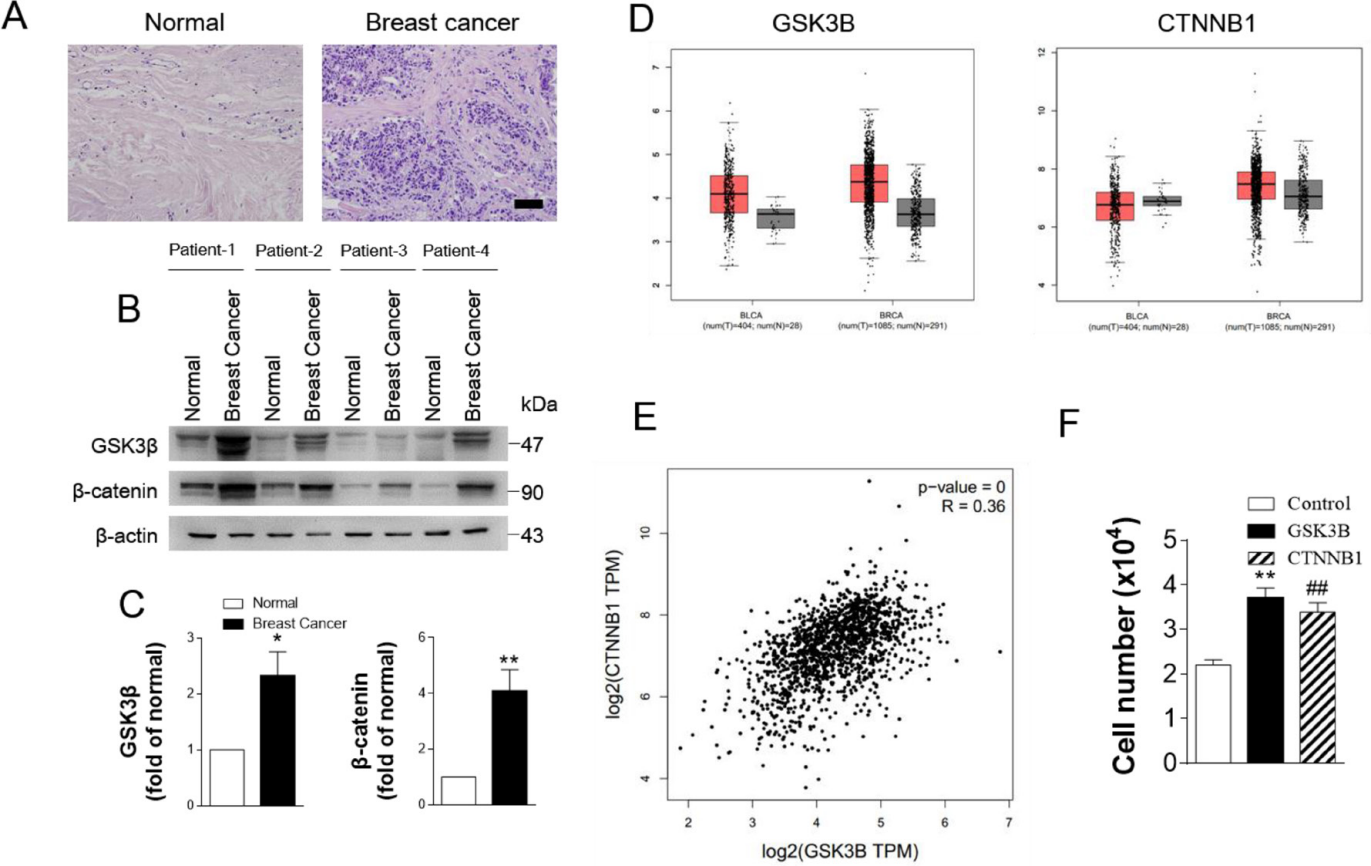
GSK3B is upregulated in breast cancer (**A**) Hematoxylin and eosin staining of breast cancer tissue. (**B**) and (**C**) Typical western blots and statistical data of GSK3B and β-catenin from normal and breast cancer tissue. *N* = 5, data are expressed as the mean ± s.e.m.; ^**^P < 0.01. ^*^P < 0.05, normal compared to breast cancer; Student’s *t*-test. (**D**) Expression level of GSK3B and CNNTB1 in cancer and normal tissues. BLCA: Bladder Carcinoma; BRCA: Breast Carcinoma. (**E**) The pair-wise gene expression correlation analysis for GSK3B and CNNTB1 in breast cancer. GSK3B and CNNTB1 are positively correlated. (**F**) Statistical analysis of cell number in MDA-MB-231 cells infected with Ad-GSK3B (MOI 25) or Ad-CTNNB1 (MOI 25). Results are expressed as mean ± SEM. ^**^p < 0.01, control *vs* Ad- GSK3B; ^##^*p <* 0.01, control *vs* Ad-CTNNB1 (*n* = 4).

**Figure 5:**
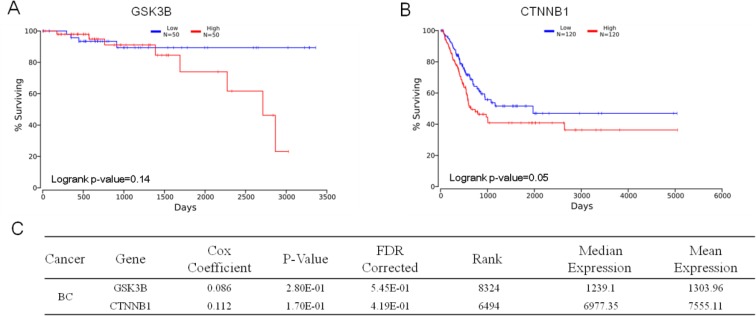
Prognostic value of GSK3B and CTNNB1 (**A**) and (**B**) Kaplan plot for GSK3B (A), CTNNB1 (B) in breast cancer. (**C**) Statistical data of GSK3B (*N* = 50) and CTNNB1 (*N* = 120). The data are expressed as the mean ± s.e.m., low expression compared to high; Student’s *t*-test.

Cross-cancer alteration analysis showed that GSK3B has an amplification pattern in most cancer types, especially in breast cancer (Figure 3). To further investigate the function of GSK3B in breast cancer, we performed immunohistochemical staining on breast cancer tissues and adjacent normal tissues. The staining results revealed significantly higher positivity for GSK3B expression in BC tissues than in adjacent normal tissues (Figure 4A). Similarly, GSK3B is also highly expressed in breast cancer patients (Figure 4B, 4C). Beta-catenin encoded by CTNNB1 is a constituent of adherens junctions and acts as an intracellular signal transducer in the Wnt signaling pathway, a pathway that is closely related with the occurrence, development, invasion and metastasis of breast cancer. We also found that β-catenin is upregulated in breast cancer patients (Figure 4B, 4C). GSK3B and CTNNB1 are highly expressed in bladder cancer and breast cancer, and they are notably positively correlated (Figure 4D, 4E). Moreover, overexpression of GSK3B or CTNNB1 increased cell proliferation (Figure 4F). High levels of GSK3B and CTNNB1 were observed in poor survival curves (Figure 5). These findings have revealed that the GSK3B signaling pathway may be a potential target for breast cancer therapy.

## DISCUSSION

As the leading cause of cancer mortality in women, BC is a serious public health problem worldwide, and the age of onset tends to be younger in recent years [1, 5, 27]. In addition, BC is lacking effective methods for early screening and diagnosis. Therefore, sensitive and specific biomarkers for BC are urgently needed. Trastuzumab, an anti-HER2 humanized antibody, has shown great clinical benefits in HER2-positive BC treatment [28]. In recent years, there has been great interest in researching the mechanisms of trastuzumab treatment. Studies have shown that numerous molecules play important roles in how a patient responds to trastuzumab, including ERBB-family SNPs, p53 protein, BAG-1 protein and individual patients’ metabolism [29–32]. Xiong *et al.* [33] confirmed that CD147 suppression enhances the effects of trastuzumab through MAPK and Akt phosphorylation while HER2 amplification level is not currently a prognostic factor for trastuzumab-based targeted therapy [34]. In addition, studies have shown that AUY922 [35] or taxane [36] plus trastuzumab is an effective regimen for patients with relapsed HER2-positive BC after (neo)adjuvant trastuzumab.

However, trastuzumab resistance has emerged as a major problem in its clinical application. Many studies have attempted to elucidate the mechanisms underlying trastuzumab resistance. Heregulin, MEOX1 and lncRNA GAS5 confers resistance to the anti-HER2 agent trastuzumab [37–39]. Some studies have demonstrated that inhibition of S100P and depletion of KLK10 results in reversal of trastuzumab-resistance (TzR) [40, 41]. In contrast to trastuzumab that inhibits the ErbB2 homodimer, another therapeutic antibody H2-18 binds to domain I of ErbB2, which induces programmed cell death (PCD) and exhibits greater antitumor efficacy than trastuzumab [42].

In the present study, we extracted data from GSE22358 and identify 1231 up-regulated and 1053 down-regulated DEGs with or without trastuzumab using bioinformatics analysis. To gain a more in-depth understanding of these DEGs, we performed GO function and KEGG pathway analysis. The GO analysis showed that up-regulated DEGs were mainly involved in DNA replication, protein N-linked glycosylation via asparagine, and response to toxic substance, and down-regulated DEGs were involved in axon guidance, protein localization to plasma membrane, protein stabilization, and protein glycosylation. Furthermore, the KEGG pathways of up-regulated DEGs included proteoglycans in cancer, the ErbB signaling pathway, bacterial invasion of epithelial cells, the insulin signaling pathway and the neurotrophin signaling pathway while the down-regulated DEGs were enriched in protein processing in the endoplasmic reticulum, base excision repair, pentose and glucuronate interconversions, and proteasome ascorbate and aldarate metabolism. This finding is consistent with the fact that glycosylation and metabolic processes play an important role in cancer processes.

We also constructed a PPI network with DEGs and listed the top eight hub genes: GSK3B, RAC1, PXN, ERBB2, HSP90AA1, FGF2, PIK3R1 and RAC2. The hub genes play an important role in cancer cell growth, migration, and invasion, especially in breast cancer [43–47]. Furthermore, these genes were involved in significant pathways, including the Fc receptor signaling pathway and regulation of the immune response pathway.

GSK3B was identified as one of the hub genes exhibiting the highest degree of connectivity. Furthermore, our experimental results showed that GSK3B was also highly expressed in breast cancer tissues and associated with poor survival (Figure 4A–4D). We hypothesize that this gene might contribute to the progression of breast cancer with trastuzumab treatment.

GSK3B associates with the destruction complex through a binding site in AXIN1 and phosphorylates β-catenin, which is subsequently targeted for proteosomal degradation [48]. Wang *et al.* [43] conducted an association study to determine whether common genetic variations in six genes (*APC, AXIN1, AXIN2, CSNK1D, CSNK1E*, and *GSK3B*) that encode the destruction complex of the Wnt/β-catenin signaling pathway account in part for the contribution of the pathway to BC risk. Mole *et al.* [49] also reported that the truncated somatostatin receptor variant sst5TMD4 is associated with increased invasiveness and aggressiveness in BC. sst5TMD4 overexpression increases vimentin, total β-catenin and phosphorylated GSK3B levels.

GSK3B can interact with β-catenin [19], and β-catenin is also highly expressed in BC. β-Catenin is a marker of poor prognosis in human cancer and has been implicated in human breast cancer, via targeting cyclin D1 or vimentin [50–52]. A number of studies have suggested that dysregulation of Wnt/β-catenin signaling occurs in human breast cancer. Blockade of Wnt/β-catenin signaling could suppress breast cancer metastasis [53, 54]. Figure 4E shows the results of the correlation analysis between GSK3B and β-catenin. GSK3B and β-catenin are notably positively correlated. Therefore, we speculated that GSK3B has a similar function as β-catenin in breast cancer, but further verification is still needed.

In conclusion, our data provide a comprehensive bioinformatics analysis of DEGs, which may be involved in the progress of trastuzumab treatment. The study provides a set of useful targets for future investigation into the molecular mechanisms and biomarkers. However, further molecular biological experiments are required to confirm the function of the identified genes.

## MATERIALS AND METHODS

### Microarray data

The gene expression profiles of GSE22358 were downloaded from the GEO database. GSE22358, which was based on the Agilent GPL5325 platform (Agilent, CA, USA), was submitted by Gluck *et al.* The GSE22358 dataset contained 154 operable early-stage BC samples receiving neoadjuvant capecitabine plus docetaxel, with (34) or without trastuzumab (120).

### Identification of DEGs

The raw data files used for the analysis included TXT files (Agilent platform). The analysis was performed using GENE-E (version 3.0, Broad Institute, USA). We applied hierarchical clustering analysis to categorize the data into two groups that had similar expression patterns with or without trastuzumab. We used a classical *t* test to identify DEGs with defined a *P* value cutoff of < 0.05 as statistically significant.

### Gene ontology and pathway enrichment analysis of DEGs

Gene ontology analysis (GO) is a common useful method for annotating genes and gene products and for identifying characteristic biological attributes of high-throughput genome or transcriptome data [55, 56]. KEGG (http://www.genome.jp/) is a knowledge base for systematic analysis of gene functions, linking genomic information with higher-order functional information [57, 58]. Comprehensively mapping a user’s gene to the relevant biological annotation in the DAVID database (https://david.ncifcrf.gov/) is an essential foundation for the success of any high-throughput functional gene analysis [59]. To analyze the DEGs at the functional level, GO enrichment and KEGG pathway analysis were performed using the DAVID online tool. *P* < 0.05 was considered statistically significant.

### Integration of a protein–protein interaction (PPI) network and module analysis

The Search Tool for the Retrieval of Interacting Genes (STRING) database is an online tool designed to evaluate protein–protein interaction (PPI) information. STRING (version 10.0) covers 9,643,763 proteins from 2031 organisms. To evaluate the interactive relationships among DEGs, we mapped the DEGs to STRING, and only experimentally validated interactions with a combined score >0.4 were selected as significant. Then, PPI networks were constructed using the Cytoscape software. The plug-in Molecular Complex Detection (MCODE) was used to screen the modules of the PPI network in Cytoscape. The criteria were set as follows: MCODE scores >3 and number of nodes >4. Moreover, the function and pathway enrichment analyses were performed for DEGs in the modules. *P* < 0.05 was considered to have significant differences.

### Integrative analysis of complex cancer genomics with cBioPortal

The cBioPortal for Cancer Genomics (http://www.cbioportal.org) provides visualization, analysis and download of large-scale cancer genomics data sets [60, 61]. GSK3B was selected for analysis in different cancers.

### Human sample collection

The patients were from Dazhou Central Hospital. (Informed written consent was obtained from all patients. The study protocol conformed to the Ethical Guidelines of the 1975 Declaration of Helsinki and was approved by the Dazhou Central Hospital Human Ethics Committee.)

### Cell culture, adenoviral infection

MDA-MB-231 cells (Cell Resource Center, IBMS, CAMS/PUMC, Beijing, China) were cultured at 37° C under 5% CO2 in Dulbecco’s modified Eagle’s medium supplemented with 10% fetal bovine serum (Gibco, Waltham, MA, USA), and 1% penicillin-streptomycin. Approximately 80% confluent MDA-MB-231were infected with adenovirus DNA expressing GSK3B or CTNNB1 at a multiplicity of infection (MOI) of 25, and grown for a further 48 h.

### Statistical analysis

All data are expressed as the mean ± s.e.m. Statistical analysis was performed with GraphPad PRISM version 5.01 (GraphPad software, Inc.) and SPSS 18.0 software package (SPSS Inc.).

## Abbreviations

BC: Breast Cancer
Bcl-xL: B-cell Lymphoma-extra Large
CC: Cell Component
CTNNB1: Catenin Beta 1
DAVID: Database for Annotation, Visualization and Integrated Discovery
DEGs: Differentially Expressed Genes
ERK1/2: Extracellular Regulated Protein Kinases
FGF2: Fibroblast Growth Factor 2
FGFR2: Fibroblast Growth Factor Receptor 2
GO: Gene Ontology
GSK3B: Glycogen Synthase Kinase 3 beta
HER2: Human Epidermal growth factor Receptor 2
HSP90AA1: Heat Shock Protein 90 Alpha Family Class A Member 1
KEGG: Kyoto Encyclopedia of Genes and Genomes pathway
KRT19: Keratin 19
MCODE: Molecular Complex Detection
Mcl-1: Myeloid Cell Leukemia 1
MF: Molecular Function
NF-κB: Nuclear Factor-κB
NUMB: Endocytic Adaptor Protein
PIK3R1: Phosphoinositide-3-Kinase Regulatory Subunit 1
PPI: Protein–Protein Interaction
RAC1: Ras-related C3 botulinum toxin substrate 1
RAC2: Ras-related C3 botulinum toxin substrate 2
RSK2: Ribosomal S6 Kinase 2
STRING: Search Tool for the Retrieval of Interacting Genes
